# New Insights into HTLV-1 Particle Structure, Assembly, and Gag-Gag Interactions in Living Cells

**DOI:** 10.3390/v3060770

**Published:** 2011-06-14

**Authors:** Keir H. Fogarty, Wei Zhang, Iwen F. Grigsby, Jolene L. Johnson, Yan Chen, Joachim D. Mueller, Louis M. Mansky

**Affiliations:** 1 Institute for Molecular Virology, University of Minnesota, Minneapolis, 18-242 Moos Tower, 515 Delaware St. SE, Minneapolis, MN 55455, USA; E-Mails: fogarty@umn.edu (K.H.F.); zhangwei@umn.edu (W.Z.); grigs011@umn.edu (I.F.G.); chen@physics.umn.edu (Y.C.); mueller@physics.umn.edu(J.D.M.); 2 Department of Diagnostic and Biological Sciences, School of Dentistry, University of Minnesota, Minneapolis, MN 55455, USA; 3 Department of Microbiology, Medical School, University of Minnesota, Minneapolis, MN 55455, USA; 4 School of Physics and Astronomy, University of Minnesota, Minneapolis, MN 55455, USA; E-Mail: john4580@umn.edu(J.L.J.); 5 Department of Biomedical Engineering, University of Minnesota, Minneapolis, MN 55455, USA

**Keywords:** deltaretrovirus, lentivirus, spectroscopy, fluorescence, tomography

## Abstract

Human T-cell leukemia virus type 1 (HTLV-1) has a reputation for being extremely difficult to study in cell culture. The challenges in propagating HTLV-1 has prevented a rigorous analysis of how these viruses replicate in cells, including the detailed steps involved in virus assembly. The details for how retrovirus particle assembly occurs are poorly understood, even for other more tractable retroviral systems. Recent studies on HTLV-1 using state-of-the-art cryo-electron microscopy and fluorescence-based biophysical approaches explored questions related to HTLV-1 particle size, Gag stoichiometry in virions, and Gag-Gag interactions in living cells. These results provided new and exciting insights into fundamental aspects of HTLV-1 particle assembly—which are distinct from those of other retroviruses, including HIV-1. The application of these and other novel biophysical approaches promise to provide exciting new insights into HTLV-1 replication.

## 1. Introduction

Human T-cell leukemia virus type 1 (HTLV-1) was the first human retrovirus identified three decades ago when it was first isolated in the United States and subsequently in Japan [1,2]. The discovery of HTLV-1 and its demonstration as the etiological agent of adult T-cell leukemia (ATL) has been previously reviewed [3–5]. HTLV-1 presently infects about 20 million individuals worldwide [6], and viral infection can also result in an inflammatory disease syndrome called HTLV-1-associated myelopathy (HAM)/tropical spastic paraparesis (TSP) [7,8]. Prevalence rates for HTLV-1 infection in the general population are greater than 1% in the Caribbean Basin, Central Africa, and South Japan [9].

## 2. HTLV-1 Replication Cycle and Virus Particle Assembly

Despite the association of HTLV-1 with cancer and its significant impact on human health, many of the details regarding HTLV-1 replication, assembly and many fundamental aspects of virus particle structure remain poorly understood. The steps involved in the replication of retroviruses such as HTLV-1 (a deltaretrovirus) include virus entry, reverse transcription of the viral RNA to DNA, integration of the viral DNA, transcription of the viral DNA, packaging of the viral RNA, particle assembly and virus particle release. Important early steps in the virus assembly pathway that are poorly understood for HTLV-1 (as well as all retroviruses) include genome recognition (Gag-RNA interactions) as well as Gag-Gag and Gag-cellular protein interactions.

Many retroviruses, including HTLV-1, initiate particle assembly in the cytosol but this does not become apparent until the accumulation of viral proteins and nucleic acid at the plasma membrane. Assembly of virus particles and budding at the plasma membrane are readily visualized by electron microscopy. Gag proteins associate with raft microdomains of the inner leaflet of the plasma membrane [10,11]. The Gag polyprotein is a primary driving force for assembly and budding. The trafficking of Gag proteins to the cell membrane and stable membrane association involve a bipartite membrane binding signal consisting of the fatty acid myristate, added cotranslationally to the N terminus of Gag in concert with a patch of basic residues within the matrix (MA) domain [12,13]. Little is known regarding details of how HTLV-1 Gag-Gag and Gag-membrane interactions are orchestrated in cells as well as the fundamental aspects for what determines Gag stoichiometry in particles and particle size.

## 3. Gag-Gag Interactions and Membrane Targeting

The difficulties in propagating HTLV-1 in cell culture precluded the thorough characterization of HTLV-1 replication, including the aspects that relate to Gag trafficking and assembly. Nonetheless, recent progress has been made using both molecular biological and biophysical techniques. Post-translation, Gag is trafficked from the cytoplasm to the inner leaflet of the plasma membrane. Gag-Gag interactions may occur either in the cytoplasm or after arrival at the plasma membrane, eventually leading to the assembly and budding of the immature virions.

Due to the paucity of knowledge concerning HTLV-1 Gag cytoplasmic behavior, it is helpful to use comparative approaches to assist in gaining greater insights into HTLV-1. The obvious candidate for such comparative analysis is HIV-1 Gag, whose trafficking and assembly pathways have been extensively studied (reviewed in [14]). In addition, HIV-1 is also a human retrovirus that, like HTLV-1, infects CD4^+^ T-cells. A relevant HIV-1 Gag assembly model is the oligomerization-dependent myristoyl-switch model, in which Gag interactions in the cytoplasm trigger the exposure of a hydrophobic myristic acid moiety which targets Gag to the inner-leaflet of the plasma membrane [15–17]. A consequence of this model is that HIV-1 Gag multimers would be depleted from the cytoplasm, as any Gag species larger than a monomer would exhibit a membrane-targeting signal. Biochemical experiments which support this model have been reported [18]. *In vitro* biochemical methods have been used to investigate HTLV-1 Gag-Gag and HTLV-1 matrix interactions in both the cytoplasmic and membrane fractions of cells, and evidence was found for Gag-Gag interactions occurring exclusively at the membrane [19]. Complimentary evidence was found by others using a variety of *in vitro* methods to investigate the self-associative behavior of the HTLV-1 Gag capsid domain. While HIV-1 capsid proteins were found to readily dimerize in solution, HTLV-1 capsid proteins exhibited no observable interactions up to millimolar concentrations [20]. These observations suggest that HTLV-1 Gag, unlike HIV-1 Gag, may initiate assembly at the inner-leaflet of the plasma membrane. Further detailed studies are needed to access this intriguing possibility.

Another model system which provides additional information concerning the possible initiation of Gag assembly in the cytoplasm is a Gag protein with a point mutation (mutation of the glycine residue near the N-terminus of Gag to alanine) that eliminates the post-translational addition of the myristic acid moiety. This G2A Gag species has been shown, for both HIV-1 [21,22] and HTLV-1 [19,23], to be lacking myristoylation and is membrane-binding deficient. The expected model for G2A mutants differs from wild-type Gag in that G2A Gag multimers are deficient in membrane-binding and thus are not depleted from the cytoplasm. Thus, G2A Gag is expected to reach higher concentrations and higher-order complexation in the cytoplasm when compared with wild-type Gag. Once again, this model has been supported by biochemical observations with the HIV-1 G2A Gag mutant [18].

It should be noted that there is ample evidence for HIV-1 Gag that myristoyl moiety exposure and subsequent membrane binding is not solely due to Gag homo-interactions. A number of studies have provided strong evidence that other factors also play a role in HIV-1 Gag membrane targeting. The lipid phosphatidylinositol (PI) 4,5-bisphosphate [PI(4,5)P_2_] has been shown in multiple studies to interact with the Gag matrix domain that favor exposure of the myristoyl moiety [24–27]. Other studies have implicated both host factors, such as calmodulin [28], and the cell environment, (pH [29]), as playing a role in myristate exposure. Such additional factors are likely to be involved in HTLV-1 Gag assembly as well. Recent studies have demonstrated that HTLV-1 Gag interacts with the inner loop of membrane-bound tetraspanins [30,31].

It is generally accepted that Gag-membrane interactions are not solely dependent on exposure of the myristic acid moiety but are also dependent on electrostatic interactions between basic residues of the matrix domain and the negatively charged phospholipid head groups of the membrane [32,33]. In addition, the viral genome has been shown to play a structural role in Gag-Gag interactions for HIV-1, and so may play a role in the initiation of Gag-Gag interactions either in the cytoplasm or at the membrane for HTLV-1 [18,34–36].

## 4. Fluorescence-Based Characterization of Gag Interactions in Living Cells

A key drawback for many of the methods used to investigate cytoplasmic HTLV-1 and HIV-1 Gag behavior is that the techniques must disrupt the cellular environment in order to isolate Gag molecules for investigation. This is problematic because the isolation of Gag molecules necessitates the removal of cellular host factors and machinery that might play a prominent role in Gag trafficking. For example, it is known the HIV-1 Gag interacts with an array of host factors on its pathway to assembly (reviewed in [14,37,38]). For this reason, it is vital to develop minimally-invasive techniques which are capable of investigating HTLV-1 Gag cytoplasmic behavior. Model systems that have used codon-optimized gag genes and carboxy-terminal tags such as GFP, have provided new tractable approaches for studying Gag trafficking and homo-interactions as well as membrane interactions. The impact of such perturbations on Gag trafficking needs further investigation.

To date, the most promising advances for studying retroviral Gag trafficking and assembly *in vivo* involve fluorescence-based techniques. These techniques typically rely on Gag constructs which are tagged with one of a number of fluorescent proteins. Fluorescence-based assays are minimally invasive, as labeled Gag proteins can be monitored in living cells which are incubated on the microscope stage. The lone perturbation to the Gag expression system is the introduction of the fluorescent tag. The introduction of a tag to the protein could have unforeseen consequences on the Gag trafficking pathway. While this is a legitimate concern, it has been shown previously that HIV-1 Gag tagged at the C-terminus with yellow fluorescent protein (EYFP) and untagged wild-type constructs are incorporated non-preferentially into virus-like particles (VLPs) [39]. VLPs are non-infectious analogues of infectious virions which contain the structural elements of the virus, but lack viral components which are required for viral propagation. They have been shown to closely mimic the structure and morphology of infectious virions (reviewed by [40]). In addition, it has been shown that HIV-1 Gag, C-terminally labeled with GFP, is incorporated into infectious virions when coexpressed with wild-type Gag [41]. Finally, a Gag construct with GFP inserted between the matrix and capsid domains of HIV-1 Gag has been shown to produce infectious viral particles with similar single-round infectivity to that of wild-type particles [42,43]. These observations support the hypothesis that the fluorescence tag may have minimal impact on Gag trafficking and assembly.

One example of a fluorescence-based technique relevant to HTLV-1 Gag behavior is imaging localization of Gag molecules in fixed cells. Studies have revealed important insights into the role of the late domain in viral assembly [44–46]. Fluorescence localization also provided evidence that HTLV-1 Gag preferentially targets tetraspanin-enriched microdomains associated with the plasma membrane for assembly [30,31], avoiding interactions with intracellular membranes [23].

**Figure 1.**
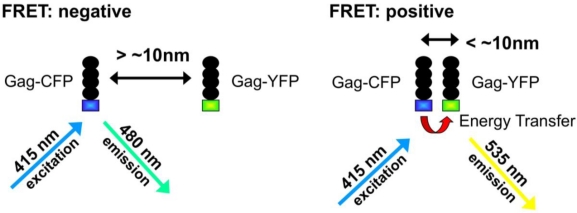
Fluorescence resonance energy transfer (FRET). FRET is used for monitoring intra- and inter-molecular interactions occurring on the nanometer scale. The transfer of excitation energy from a “donor” fluorophore (Gag-CFP) to an “acceptor’ fluorophore (Gag-YFP) is monitored. The donor molecule is excited with light at a wavelength that does not excite the acceptor molecule. At distances between donor and acceptor greater than approximately 5 nm, the excited donor molecule emits fluorescence at its characteristic wavelength. At distances below 5 nm, the excited donor molecule can transfer energy to the acceptor molecule, resulting in the acceptor fluorescing at a longer wavelength. In this example, a decrease in Gag-CFP signal, with a corresponding increase in Gag-YFP signal would indicate Gag-Gag interactions.

Another promising technique for the *in vivo* study of retroviral Gag trafficking and assembly is fluorescence resonance energy transfer (FRET) (reviewed in [47,48]) (Figure 1). This technique relies on energy transfer between two different fluorophores (an energy donor and an energy acceptor) to probe intra- and intermolecular interactions. The efficiency of energy transfer depends on the inverse sixth power of the distance between the fluorophores, and is used to monitor interactions at the nanometer scale. FRET has been used to probe Gag-Gag interactions for HIV-1 [49,50] and RSV [51], as well as HIV-1 Gag-nucleic acid interactions [36] and HTLV-1 NC-nucleic acid interactions [52].

The fluorescence techniques described so far provide a suitable technology platform for the investigation of retroviral Gag trafficking and assembly in living cells. In recent years, the development of a suite of biophysical fluorescence techniques has further augmented the analytical power of fluorescence-based investigations. In particular, total internal reflection fluorescence (TIRF) (reviewed by [53]) and two-photon fluorescence fluctuation spectroscopy (FFS) (reviewed by [54,55]) have been used to reveal intriguing insights into Gag behavior.

**Figure 2.**
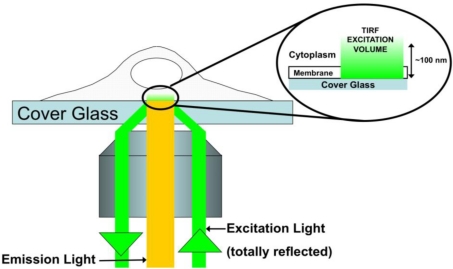
Total Internal Reflection Fluorescence (TIRF) microscopy. In objective-based TIRF microscopy, the excitation laser is positioned to enter at the edge of the back aperture of a high-NA objective (NA ≥ 1.45). The beam emerges from the objective at an angle, which is greater than the critical angle of the cover-slip/sample interface. Thus, the excitation beam is totally reflected back down through the objective. Despite this total reflection, an evanescent, or near-field, wave penetrates approximately 100 nm into the sample (inset). This results in a spatially confined excitation region at the cover-slip/sample interface. In cellular applications, the near-field light would only excite fluorophores at the bottom membrane, and in a small region of the cytoplasm proximal to the membrane. Therefore, this technique is ideal for probing proteins that interact with the membrane.

In TIRF microscopy, excitation light is transmitted to the cover slip-sample interface at an angle greater than the critical angle (Figure 2). This results in the incident light being totally reflected at the interface. Despite the total reflection of the excitation light, an evanescent field is established that penetrates into the sample to a depth of about 100 nm. In retroviral Gag *in vivo* studies, this excitation penetration depth corresponds to the cell plasma membrane and the cytoplasm-membrane interface. The limited penetration of the excitation light only excites fluorophores at or proximal to the membrane, and therefore greatly increases sensitivity and signal to noise due to the elimination of cytoplasmic background fluorescence. Thus TIRF microscopy is ideal for studying the steps of Gag trafficking and assembly which occur at the plasma membrane.

To date, TIRF studies have focused on HIV-1 Gag behavior. Recent studies monitored the dynamics of VLP assembly at the membrane, determining the timescale from the beginning of detectable assembly at the membrane (∼10 Gag molecules) to eventual budding. Jouvenet *et al.* determined that 5–6 minutes passed from the first detection of an assembling VLP to a steady-state assumed to be the assembly endpoint [56]. In another study, Ivanchenko *et al.* demonstrated that the steady-state observed in the previous study was likely the budding of the viral particle and is followed by VLP release [57]. They found that the average timescale for HIV-1 VLP assembly, budding, and release was closer to 25 minutes. In a more recent study, the role that the viral genome plays in Gag assembly at the membrane was investigated [35]. It was found that RNA is detectable at a membrane assembly site before Gag is present at detectable levels, supporting the idea that viral RNA plays a structural role in VLP assembly. Finally, a recent study examined the role of the cellular ESCRT machinery in HIV-1 budding and release (For the role of ESCRT complexes in HIV-1 budding and release, see [41]). The study focused on the ESCRT-associated protein VPS4A, which is thought to play a role in membrane scission, and thus particle release. The study found that VPS4A proteins indeed arrive at sites of fully-assembled VLPs, promoting VLP membrane scission and release on a ∼35 s timescale [58].

**Figure 3.**
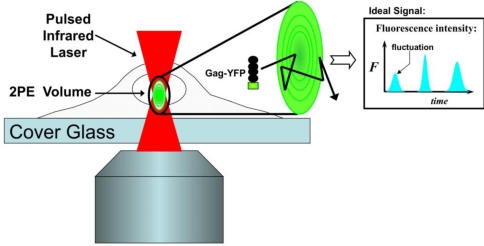
Fluorescence Fluctuation Spectroscopy (FFS). FFS monitors the fluorescence of single molecules moving through a laser excitation region. In FFS using two-photon excitation (2PE) a pulsed infrared laser is used to create a small excitation region (∼1/1000^th^ the volume of a cell) in the sample. The fluorophore is only excited in the focal region of the beam, as explained in the text. A single Gag-YFP molecule which either diffuses or is transported through the focal volume (inset) causes a burst in detected fluorescent intensity. By monitoring the characteristic timescale, amplitude, and frequency of the fluorescent fluctuations caused by single molecules moving through the focus, one can obtain information relating to protein mobility (duration of fluctuations), protein stoichiometry (amplitude of fluctuations), and concentration (frequency of fluctuations).

Two-photon fluorescence fluctuation spectroscopy (FFS) is a quantitative method which is ideally suited to explore protein interactions in living cells, and has been used to study RSV [51], HIV-1 [39,59], and HTLV-1 [59,60] Gag behavior (Figure 3). In two-photon excitation, a pulsed infrared laser, approximately double the one-photon excitation wavelength of the fluorescent protein, is focused within the sample of interest. If the laser intensity is high enough at the focal position, the fluorophore can be excited by the near simultaneous absorption of two infrared photons, each supplying half of the total excitation energy [61]. Two-photon excitation is restricted to the close proximity of the laser focus, because it is only in this region that the intensity is high enough to support efficient two-photon absorption. Knowledge of the optics allows the calculation of a focal volume ([62]), which provides a convenient measure of the effective volume observed by two-photon excitation. This results in fluorophore excitation which is limited to a spatially defined volume on the order of less than one femtoliter (10^−15^ L, ∼1/1000th of a cell’s volume). This spatially defined excitation method has distinct advantages over one-photon excitation: it minimizes photodamage to the small two-photon excitation volume and thus minimizes photobleaching of biological samples. In addition, it avoids potential contamination of single molecule fluorescence from Raman scatter of the solvent [61,63].

FFS monitors the fluorescence fluctuations caused by single-labeled proteins that migrate in and out of the diffraction-limited two-photon observation volume. Analysis of the fluctuations provides information on the concentration, mobility, and brightness of fluorescent proteins [62,64,65]. The most common application of FFS analysis is the investigation of protein diffusion and transport through investigation of fluorescence burst duration, or correlation analysis. FFS restricted to correlation analysis is also known as fluorescence correlation spectroscopy (FCS), and was introduced by Webb and coworkers [66,67]. Determination of protein stoichiometry through brightness analysis, or investigation of fluorescence fluctuation amplitude, is another analysis technique used in FFS. This brightness-based method uses the photon counting histogram (PCH) to determine the number of molecules in the observation volume and their brightness from the shape of the photon count distribution [68]. Brightness is a measure of the average fluorescence intensity of a single molecule passing through the observation volume, and is reported in counts per second per molecule (cpsm). Access to the brightness of fluorescent proteins provides a unique way to determine the stoichiometry of proteins in cells. A monomer of Gag, labeled with a fluorescent protein, has a certain characteristic brightness, based on the nature of the fluorophore. A dimer of labeled Gag molecules has two, identical fluorophores, and thus would be twice as bright, with higher-order complexes being higher integer multiples of monomer brightness. Brightness has been shown to be a robust technique for the determination of protein stoichiometry over a wide range of oligomerization levels [39,60,64].

The single molecule sensitivity and high spatial-temporal resolution of FFS allows for the comprehensive study of the Gag trafficking and assembly pathway. This is evident in recent FFS studies used to characterize RSV, HIV-1, and HTLV-1 Gag behavior.

## 5. Cryo-Electron Microscopy (Cryo-EM) and Cryo-Electron Tomography (Cryo-ET) of Retroviral Particles

**Figure 4.**
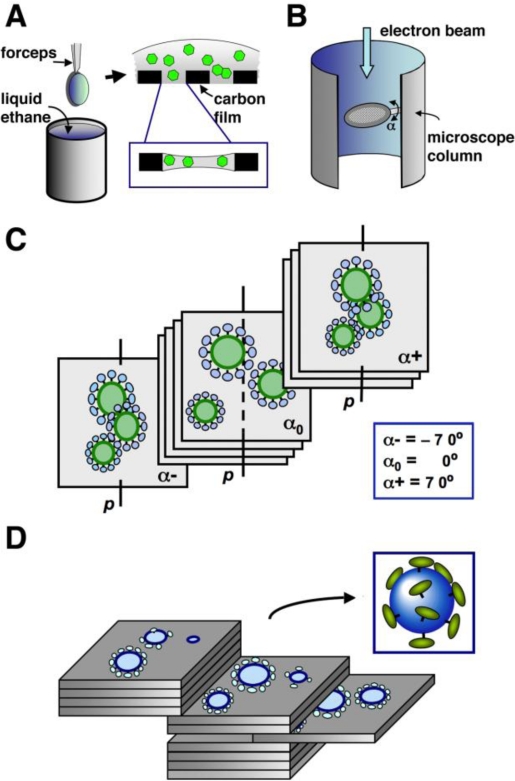
Cyro-electron microscopy (cryo-EM) and cryo-electron tomography (cryo-ET). (**A**) Preparation of frozen hydrated cryo-EM grid. A small aliquot (∼3 μL) of specimen is added to a perforated carbon TEM grid. The grid is blotted by a filter paper and is quickly plunged into liquid ethane. The water in the specimen forms vitreous ice that is suspended across the holes of the perforated carbon film. The inset panel shows virus particles (green hexagons) embedded in the vitreous ice (grey). (**B**) Position of the TEM grid relative to the electron microscope column. The specimen holder in the microscope is perpendicular to the electron beam. The TEM grid can be rotated between −70° to 70° (α angle) around the tilt axis of the specimen holder. (**C**) A diagram showing a set of images of three virus particles (green) recorded at various α tilt angles (inset). “p” represents the position of the specimen holder tilt axis in each image. (**D**) Three-dimensional (3D) reconstruction map and an extracted model of one virus particle. The reconstruction map is represented as a set of two-dimensional slices, which cut through the 3D map of the virus particles (blue). The 3D model of any virion (inset) can be extracted from the reconstruction map for further analysis.

The cryo-EM and three-dimensional (3D) reconstruction technique is a powerful tool in determining the structure of a broad spectrum of samples ranging from 200 kDa macromolecules to large cellular organelles and small cells in their native frozen-hydrated state [69–71]. Cryo-EM allows the study of structures of specimens that do not form well-diffracted crystals or molecular complexes that are too large for NMR spectroscopy. Interesting molecular activities can be arrested when the specimen is frozen in liquid ethane (∼−180 °C) (Figure 4A) and molecular structural details are preserved with minimal artifacts. With the employment of the field emission gun electron microscope and advancement of robust computational software, the resolution of a cryo-EM map for specimens such as icosahedral viruses and symmetric protein complexes is achievable to near atomic level [72–76]. Given these advances, cryo-EM has become an indispensable tool in modern structural biology [77].

Because retroviruses have heterogeneous morphology, structural investigation of retrovirus particles has traditionally relied on electron tomography (ET) [70,78]. ET studies the structure of one-of-a-kind objects, such as cellular organelles [79–81], retroviruses [82–85] and small cells [86–88]. Shown in Figure 4B–C, ET involves recording images of the same specimen area at various tilt angles in the microscope (within ±70°). The recorded tilt series are then translationally and rotationally aligned to be brought into register for computation of a 3D reconstruction map (Figure 4D) [89]. The alignment is accomplished by calculating either the cross-correlation of images at close tilt angles [90–92] or the shift vectors and rotation using colloidal gold particles mixed with the specimen [93–95]. The 3D reconstruction map is then calculated using a weighted back projection method [96] or algebraic iterative techniques [97,98]. Due to the limitation of the tilt angles of the specimen stage in the microscope, the tomography data has missing regions in space. The incompleteness of the data and high electron radiation damage of the frozen specimen limit the attainable resolution of the ET and cryo-ET reconstruction maps to 4–6 nm [99,100].

When compared to x-ray crystallography and NMR spectroscopy, cryo-EM and cryo-ET reconstruction methods provide reconstruction maps at lower resolution. The sub-tomogram averaging method [101,102] is used for improving the resolution of a sub-volume in the reconstruction map that allows fitting of the atomic structure of the protein components into the boundary of the electron densities of the macromolecular complexes [82,85,103]. Understanding molecular organization of the proteins helps to derive the proteins boundary and molecular interface, so the key amino acid residues which are essential to the virus assembly and function can be identified. The sub-tomogram averaging technique has been utilized in many studies that determine the Gag-Gag assembly in immature retrovirus particles [82,104–106].

Extensive cryo-ET studies on retroviral assembly and maturation have been focused on immature and mature virus-like particles (VLP) of HIV-1 [82,83,104,105,107], Rous sarcoma virus (RSV, an alpharetrovirus) [84,108,109] and Mason-Pfizer monkey virus (MPMV, a betaretrovirus) [106]. In HIV-1 immature particles, it was found that the capsid proteins (CA) are arranged in a single, continuous but incomplete hexameric lattice with irregular defects and about 8 nm spacing between the C-terminal domains of CA [82,107]. Similar Gag-Gag arrangements in immature particles were also demonstrated in RSV and MPMV through cryo-ET reconstruction and sub-tomogram averaging methods, suggesting the molecular organization of Gag are conserved among several retrovirus genera [106]. Although there is very little sequence similarity, the tertiary structures of the matrix (MA), CA and nucleocapsid (NC) domains of Gag are conserved among the retroviruses [110]. The conserved Gag lattice found in three genera of retroviruses implies that intermolecular interactions of these domains in Gag are critical during assembly of the retrovirus immature particles.

Understanding the structure of mature retroviruses includes investigating the organization of the cores that encapsulate the viral genome and the arrangement of the viral envelope proteins using cryo-EM and cryo-ET reconstruction methods. During retrovirus maturation, proteolytic cleavage of Gag leads to rearrangement of CA and formation of the retroviral core. The nucleocapsid domain of Gag and the viral RNA are condensed into a ribonucleoprotein complex which is encapsulated inside the core. The matrix domain of Gag remains associated with the viral membrane. The cone-shaped capsid is composed of pentameric and hexameric arrangements of CA subunits. *In vitro*, the HIV-1 CA spontaneously assembles into helical tubular structure and cone-shaped particles that resemble authentic viral capsids [111]. The helical tubular structure of CA allows determination of the hexameric arrangement of CA at high resolution. Fitting the atomic structures of CA into the reconstruction map demonstrated that the N-terminal domain of CA forms hexameric rings and the C-terminal dimerization domains connect the ring-structures together. A more recent study [112] using a 16 Å resolution cryo-EM reconstruction map of a tubular-assembled HIV-1 CA in conjunction with an atomic-resolution NMR structure of the C-terminal domain dimer of CA reviewed critical amino acids residues at the CA protein interface. Understanding of the pentameric arrangements of CA is derived from cryo-EM reconstruction of *in vitro*-assembled capsids of RSV that have icosahedral symmetry [109]. This study suggested that electrostatic interactions govern the differential assembly of pentamers and hexamers in retrovirus cores.

Cryo-ET reconstruction and sub-tomogram averaging methods were also used to study the trimeric HIV-1 envelope protein spikes and its interaction with neutralizing antibodies and cell surface receptor CD4 [85,103]. Each envelope protein spike is composed of three copies of a heterodimer of glycoproteins gp41 and gp120. Molecular models for the native HIV-1 gp120 trimers in unbound particles and the CD4-bound states derived from the study suggested that binding of CD4 introduces a major reorganization of the gp41 and gp120 monomers in HIV-1 spike proteins that leads to closer contact between the viral and target cell membranes [85].

The ultimate goal of studying the structures of immature and mature retroviral particles is to better understand the molecular mechanisms that drive virus assembly, budding, maturation and infection. Cryo-ET studies on the HIV-1 budding sites in HeLa and MT-4 cells [113] implicate that budding is initiated by Gag assembly and is completed in an ESCRT-dependent manner before the Gag sphere is complete. In a follow-up study [114], the structure of the Gag lattice of VLPs at the budding site in human cells was found to be indistinguishable from those of released immature VLPs, suggesting little structural rearrangement of Gag proteins occur after virus budding and before proteolytic cleavage. Another study spearheaded investigation of the virological synapse presented in HTLV-1 immortalized cells MS9 [115] using ET of plastic thin sections combined with immunostaining of the viral proteins. The study illustrated in 3D the synaptic clefts that have HTLV-1 particles and are surrounded by the tightly apposed plasma membranes of two cells. The size of the synaptic clefts in the reconstruction map suggested that HTLV-1 virions can contact the recipient cell membrane before detaching from the infected cell.

Cryo-EM, ET and cryo-ET studies on retroviral VLPs (especially HIV-1), in conjunction with the x-ray crystallography and NMR studies of individual domains of Gag proteins [17,116–124] have provided vast structural information on viral structural protein organization in immature and mature particles of various retroviruses and retroviral VLPs in the context of cellular environment. Application of these techniques to HTLV-1 is still very limited [60,115,125]. We expect to see exciting progress in this field in the near future.

## 6. FFS and Cryo-EM Characterization of HTLV-1 and HIV-1 Gag

The first application of FFS to a retroviral Gag system was the use of FRET and FFS to investigate both RSV Gag-Gag interactions, and cytoplasmic Gag mobility [51]. FRET was used to demonstrate that Gag-Gag interactions were occurring both at the membrane and in the cytoplasm. FFS analysis investigated the mobility and brightness of cytoplasmic Gag, and found that Gag exhibited mobility indicative of large protein complexes, while the brightness indicated that the Gag stoichiometry in these complexes was low, and thus it was likely that unlabeled host proteins were present in the complexes.

FFS was subsequently applied to the analysis of Gag stoichiometry and size by brightness and FCS analysis for both HIV-1 [39,126] and HTLV-1 [60] virus-like particles. It was found that HIV-1 Gag stoichiometry ranged from 750 to 4000 copies of Gag per VLP, with an average VLP diameter of 130 nm. While the Gag copy number per VLP varied, the size remained constant. This led to the proposed hypothesis that Gag formed an incomplete shell in VLPs. This hypothesis was confirmed independently by cryo-ET. The average size of HIV-1 particles reported by FFS and cryo-EM agree with each other. The stoichiometry derived from cryo-EM studies of both immature and mature HIV-1 particles overlaps with the stoichiometry range reported by FFS. [82,83,111].

Recently a study by Grigsby *et al.* applied both FFS and Cryo-EM towards the study of HTLV-1 particles [60]. This work provided a first glimpse into the structure, stoichiometry and size of HTLV-1 viral particles. A key component of this study was the development of a codon-optimized HTLV-1 Gag-EYFP construct which overcame the challenge of HTLV-1’s low expression levels in cell culture. In this study, Cryo-EM was used to investigate the size and morphology of HTLV-1 VLPs, while FFS was implemented for both size and Gag stoichiometry information. It was found that HTLV-1 virus-like particles were significantly smaller than HIV-1 VLPs, the particle diameters found were 71 ± 20 and 75 ± 4 nm by Cryo-EM and FFS respectively. This is a little more than half the particle diameters found by both FFS and Cryo-EM for HIV-1 viral particles. The smaller size found for the particles was complimented with a decrease in the average HTLV-1 Gag stoichiometry of VLPs, relative to HIV-1 particles. The average Gag stoichiometry found for HTLV-1 VLPs was ∼500 copies per particle. This number supports the hypothesis that, like HIV-1 VLPs, there is a partial Gag shell in the average HTLV-1 VLP, as a fully closed shell would be expected to have a Gag stoichiometry of ∼1300.

The VLP studies confirmed FFS as a technique that could independently verify size and stoichiometry information obtained by Cryo-EM. In addition, the sample preparation of VLPs for FFS analysis is extremely simple, as VLPs can be obtained directly from the culture supernatant, without the need for concentration, purification, and careful preparation required by Cryo-EM. While FFS cannot match the kind of structural information available to Cryo-EM, its ability to perform quantitative analysis in living cells renders it a powerful tool in the study of HTLV-1.

A more recent FFS study of Gag trafficking and assembly investigated the extent of both HIV-1 and HTLV-1 Gag oligomerization in the cytoplasm [59]. The average cytoplasmic stoichiometry of Gag *versus* cellular expression level was found for both the wild-type species and the respective G2A mutants. It was found that, for both HIV-1 and HTLV-1 wild-type species, Gag was predominantly monomeric over the full range of measured cytoplasmic concentrations (10 nM to ∼600 nM). The data for HIV-1 wild-type Gag agrees well with a recent study, in which the isolated, cytoplasmic fraction of HIV-1 Gag was found to be dominated by Gag monomers [18]. A unique aspect of FFS is its ability to explore the cytoplasmic oligomerization-state of Gag *in vivo*, on a cell-by-cell basis, as a function of the cellular expression level.

Investigation of cytoplasmic G2A mutants for both HIV-1 and HTLV-1 by FFS revealed intriguing differences in the behavior of the two Gag species. HIV-1 G2A Gag exhibited a concentration-dependent increase in its average oligomerization (from monomers to greater than octamers) over a concentration range from 10 nM to ∼6 μM. Again, these findings are in agreement with a recent study on HIV-1 [18], although it has also been reported that myristoylation is required for HIV-1 multimerization [127]. This discrepancy may indicate that Gag-Gag interactions occurring in the cytoplasm are relatively weak.

This data supports the model for HIV-1 Gag assembly and membrane targeting where Gag interactions in the cytoplasm lead directly to myristate exposure and membrane targeting. For wild-type, Gag oligomers are depleted from the cytoplasm, and thus the dominant cytoplasmic Gag species is the monomer. In addition, depletion of Gag from the cytoplasm caused by targeting to the membrane places an upper limit on the cytoplasmic concentration of Gag. G2A mutants lack the myristoyl moiety which promotes Gag membrane association. This results in a Gag species that is strictly cytoplasmic. G2A Gag oligomers do not have a sufficient membrane-binding signal and thus remain in the cytoplasm. As a consequence, G2A Gag accumulates to much higher concentrations in the cytoplasm when compared to wildtype, which favors the formation of oligomerized Gag species.

Surprisingly, HTLV-1 G2A Gag exhibited no increase in oligomeric state, despite reaching the same elevated cytoplasmic concentrations found for HIV-1. A previous study by Rayne and coworkers corroborates this evidence as they found no evidence for G2A Gag complexes in the cytoplasmic fraction of lysed cells [19]. This difference in the behavior of the two viral Gag species suggests that the HTLV-1 Gag trafficking pathway might differ from HIV-1. It was proposed by Rayne and coworkers that myristoylation is required for HTLV-1 Gag multimerization. The FFS data corroborates the evidence for a possible difference in the location of the onset of Gag-Gag interactions for HIV-1 and HTLV-1, respectively.

The FFS study which probed HTLV-1 and HIV-1 Gag-Gag interactions in the cytoplasm also discovered some interesting information concerning the behavior of membrane-bound Gag for both species [59]. To ensure that cytoplasmic fluorescence measurements were not altered by fluorescence from Gag complexes at the membrane, the study made use of the recently developed method of z-scan FFS [128–130]. In z-scan FFS, the two-photon excitation volume is scanned through the cell along the z-axis, entering the cell through the membrane-medium interface, and exiting through the membrane-coverslip interface. This z-scan yields a fluorescent intensity trace which contains information concerning both the geometry of the cell and the size of the focal volume [128]. In cells expressing Gag, there are populations of Gag molecules heterogeneously distributed both at the membrane and in the cytoplasm. At locations where there is a population of Gag at the membrane, the z-scan fluorescent intensity profile identifies the presence of membrane-bound Gag. The z-scan FFS profile can be analyzed to determine the nature of the Gag membrane-bound fraction at precise positions within a cell.

The z-scan FFS analysis applied to the HTLV-1 and HIV-1 Gag systems found some interesting aspects in the behavior of membrane-bound Gag species for both viruses. It was found that, for both viruses, a significant portion of the surface of the plasma membrane was covered by non-punctate Gag with a low degree of oligomerization. Thus, Gag is not present at the membrane solely as discrete puncta, but also as a kind of dynamic “sheet” of low copy number Gag complexes. Time-resolved z-scan measurements found that the presence of membrane-bound HIV-1 and HTLV-1 Gag at measured positions was long-lived; the persistence of the membrane-bound fraction exceeded the duration of the experiments, which monitored the membrane for 20 minutes or more. The apparent concentration of Gag at these positions was dynamic; membrane intensity fluctuations implied mobile, low order Gag complexes which change the local surface density of Gag at the membrane with time.

## 7. Emerging Fluorescent Methodologies

The application of biophysical fluorescence approaches to the study of retroviral Gag trafficking and assembly has provided novel information concerning not only HTLV-1 Gag behavior, but also for the comparatively well-studied HIV-1 Gag species. Quantitative, time-resolved analysis of Gag behavior at the membrane has been achieved with near single molecule resolution using TIRF microscopy [35,56–58]. FFS has been demonstrated to have unparalleled sensitivity for quantifying retroviral Gag behavior, from the cytoplasmic behavior of mostly monomeric Gag [51,59] to the stoichiometry and size of retroviral VLPs [39,60].

There are a number of recently developed biophysical fluorescence techniques which hold promise for application to the study of HTLV-1 Gag trafficking and assembly. Perhaps the most exciting advances in fluorescence microscopy in recent years are sub-diffraction fluorescence imaging techniques (reviewed by [131]). In conventional fluorescence microscopy, the optical resolution of the instrument is diffraction limited to about half the wavelength of the excitation light. For instance, for excitation light with a wavelength of 500 nm, the highest achievable resolution of a fluorescence experiment would be 200 nm. In other words, if two fluorescent proteins are separated by less than 200 nm, they are indistinguishable from one another in conventional light microscopy. As a single virion of HTLV-1 or HIV-1 is actually smaller than this resolution limit, structural or morphological features of the virion are unobservable by conventional fluorescence microscopy. In recent years, however, an array of techniques have been developed that take advantage of both the unique properties of fluorophores and the physics of light to accurately obtain resolution down to tens of nanometers. As a whole, these techniques are referred to as super-resolution microscopy (reviewed by [131,132]).

One category of super-resolution spectroscopy uses the idea of reversible switching of fluorophores between an ‘on’ and ‘off’ state in order to limit the fluorescence to a sub-diffraction limited volume. The most common example is stimulated emission depletion spectroscopy (STED) [133,134]. In this technique, two laser beams are used. One laser is the excitation laser, which is at the excitation wavelength of the fluorophore. The second beam is used for stimulated emission depletion; it is at a wavelength which causes stimulated emission of the fluorophores at the wavelength of the depletion laser. This stimulated emission forces the fluorophores back to the ground state. The excitation is focused to a diffraction limited spot, while the depletion laser is focused to a diffraction limited “donut” at the same position of the excitation spot. The “donut hole” of the depletion focus, which is much smaller than the excitation spot, is the only position at which molecules fluoresce, because it is the only region at which the excitation beam is not overlapped by the depletion beam.

**Figure 5.**
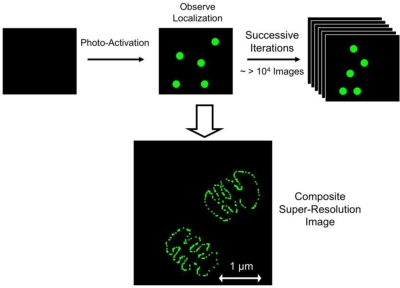
Super-resolution microscopy. Super-resolution microscopy obtains fluorescence-based spatial information with sub-diffraction (<200 nm) resolution. This is accomplished by using photo-activatable (PALM/fPALM) or photo-switchable (STORM) fluorophores, such that a sparse, spatially-separated sub-population of fluorophores is active at any one time. Upon photo-activation of the sub-population, the positions of the well-separated fluorophores can be determined with high spatial resolution by mapping the center of the diffraction-limited fluorescence signal. The activated fluorophores are then de-activated either by photobleaching (PALM/fPALM) or photo-switching, and a different sub-population is activated and localized. By repeating this process thousands of times, a composite, super-resolution image can be constructed using the mapped position of every fluorophore.

A second category of super-resolution involves the photoswitching or photoactivation of fluorophores, such that only a sparse few are fluorescing at any given time. If the fluorescing molecules are positioned such that there is only one fluorophore located within a diffraction-limited spot, their relative positions can be found with nanometer-scale resolution. In the next phase of the experiment, a different subset of fluorophores is “turned on” either through a photoswitching or photoactivation process, such that another distinct subset of fluorophores can be precisely located within the image. By switching fluorophores “on” and “off” through several iterations, a composite image with nanoscale resolution can be reconstructed from the multiple images of sparsely activated fluorophores. Super-resolution microscopy which relies on stochastic photoswitching of fluorophores is known by the name stochastic optical reconstruction microscopy (STORM) [135] (Figure 5). Super-resolution microscopy which relies on photoactivation of fluorophores is referred to as photoactivation localization microscopy (PALM) [136] or fluorescence photoactivation localization depletion (fPALM) [137].

A recent application of super-resolution PALM microscopy to HIV-1 Gag molecules at the cellular membrane provided unique insights into membrane-bound behavior [138]. The authors developed a single-particle tracking PALM methodology (stPALM) which allowed the dynamic tracking of individual Gag molecules at the membrane of COS7 cells. It was found that ∼67% of Gag molecules tracked were located within clusters of Gag molecules, consisting of at least five Gag molecules residing in an area of 300 nm. This demonstrated the resolvability of single Gag molecules within putative sites of VLP assembly, something unachievable with conventional microscopy techniques.

## 8. Summary

Two key recent advances provide much excitement in learning more about the detailed mechanisms of HTLV-1 replication, particularly those involving virus assembly, release as well as particle structure and morphology. First, the development of tractable model systems has vastly improved the ability to conduct tissue culture-based experimentation on HTLV-1 replication. Second, the use of state-of-the-art biophysical technologies has tremendous promise in enhancing the ability to quantitatively investigate HTLV-1 virus assembly and release. The application of these and other exciting technologies under development should lead to many significant discoveries in our understanding of these and other aspects of HTLV-1 biology, which may provide new insights into virus control.
